# Uma articulação conceitual para boas práticas preventivas (ou para a prevenção quaternária)

**DOI:** 10.1590/0102-311XPT068123

**Published:** 2024-09-16

**Authors:** Charles Dalcanale Tesser

**Affiliations:** 1 Centro de Ciências da Saúde, Universidade Federal de Santa Catarina, Florianópolis, Brasil.

**Keywords:** Prevenção de Doenças, Prevenção Primária, Prevenção Secundária, Prevenção Quaternária, Educação Médica, Disease Prevention, Primary Prevention, Secondary Prevention, Quaternary Prevention, Medical Education, Prevención de Enfermedades, Prevención Primaria, Prevención Secundaria, Prevención Cuaternaria, Educación Médica

## Abstract

A prevenção é consensualmente defendida, especialmente para as doenças crônicas não transmissíveis. Porém, dada a proliferação de tecnologias preventivas, não parece defensável estimular genericamente condutas e exames preventivos em profissionais de saúde e usuários. Neste ensaio, apresentamos uma articulação de conceitos, ideias e critérios para a consideração de medidas preventivas, como um roteiro mínimo a ser manejado pelos profissionais (especialmente os da atenção primária à saúde) e gestores. São articulados os conceitos de: prevenção primária, secundária e quaternária; prevenção redutiva e aditiva, estratégias preventivas de alto risco e populacional; medicina baseada em evidências e sua crise contemporânea; princípio da precaução; promoção da saúde, abordagem ampliada e centrada na pessoa e decisão compartilhada. Tal articulação foi concebida visando melhorar a competência na avaliação de medidas preventivas, tornando as decisões clínicas e sanitárias mais criteriosas e menos iatrogênicas quanto à prevenção primária e secundária.

## Abstract (en)

Prevention is universally advocated, especially in the case of noncommunicable diseases. However, given the proliferation of preventive technologies, it does not seem defensible to generically encourage preventive behaviors and tests for healthcare professionals and users. In this essay, we articulate concepts, ideas and criteria for considering preventive measures, providing a minimum guide to be used by professionals (especially in primary healthcare) and managers. The concepts of primary, secondary and quaternary prevention are explored, as well as those of reductive and additive prevention, high-risk and population-based preventive strategies; evidence-based medicine and its contemporary crisis; the precautionary principle; health promotion, an expanded, person-centered approach and shared decision-making. This discussion was designed to improve competence in the evaluation of preventive measures, making clinical and health decisions more judicious and less iatrogenic regarding primary and secondary prevention.

## Abstract (es)

La prevención es defendida consensuadamente, especialmente en las enfermedades crónicas no transmisibles. Sin embargo, dada la proliferación de tecnologías preventivas, no parece sostenible fomentar genéricamente conductas y exámenes preventivos entre los profesionales de la salud y los usuarios. En este ensayo, presentamos una articulación de conceptos, ideas y criterios para considerar medidas preventivas, como una guía mínima que manejar por parte de los profesionales (especialmente en la atención primaria de salud) y gestores. Se articulan los conceptos de prevención primaria, secundaria y cuaternaria; prevención reductiva y aditiva, estrategias preventivas de alto riesgo y poblacional; medicina basada en evidencias y su crisis contemporánea; principio de precaución; promoción de la salud, abordaje ampliado y centrado en la persona y toma de decisiones compartida. Esta articulación fue diseñada para mejorar la competencia en la evaluación de medidas preventivas, haciendo que las decisiones clínicas y de salud sean más juiciosas y menos iatrogénicas con relación a la prevención primaria y secundaria.

## Introdução

É comum que gestores, médicos e outros profissionais da saúde operem com noções sobre prevenção e atitudes positivas relativas às ações preventivas, sobretudo em doenças crônicas e especialmente nas não transmissíveis como cânceres e doenças cardiovasculares, que são as maiores causas de morbimortalidade no Brasil. Os conceitos usados geralmente envolvem prevenção primária (P1: intervenções preventivas antes do estabelecimento de uma doença, agravo ou situação a ser prevenida), prevenção secundária (P2: intervenções para identificar e tratar precocemente uma doença ainda assintomática, visando reduzir sua morbimortalidade) e terciária (P3: reabilitar e prevenir complicações de doenças com lesões já estabelecidas), de Leavell & Clark ^1^, e pouco mais que isso.

Boa parte das práticas clínicas preventivas podem ser resumidas a solicitar exames e orientar condutas aos usuários, e exortá-los a segui-las. Subjacente a tal mensagem está um apelo moral e ético otimista e afirmativo de que ações preventivas são desejáveis e necessárias. Com a transição epidemiológica brasileira, ainda que com uma tripla carga de doenças - alta morbimortalidade por doenças crônicas não transmissíveis coexistindo com uma elevada incidência e prevalência de doenças infecto-parasitárias, sobretudo no primeiro decênio, e de causas externas, principalmente homicídios, na população masculina jovem (15-29 anos) ^2^ -, esse apelo preventivo foi amplificado até se tornar uma obrigação moral ^3^ ^,^ ^4^, e também foi reforçado pelo discurso da nova promoção da saúde, que a ele se associou ^5^. Por fim, a proliferação e sofisticação de tecnologias preventivas diagnósticas e terapêuticas operacionalizou tal apelo e impulsionou o crescimento das ações preventivas na clínica ^4^, dando consequência tardia ao movimento da medicina preventiva, defensor de uma atitude preventiva nos médicos focada no indivíduo e sua família ^6^. Hoje, no Brasil e fora dele, cuidados preventivos são um dos mais frequentes motivos de consulta médica na atenção primária à saúde (APS) ^7^ ^,^ ^8^.

Todavia, a proliferação de ações clínicas preventivas pouco criteriosas pode gerar mais danos que benefícios às pessoas e produzir iniquidades - diferenças desnecessárias, evitáveis e injustas moral e socialmente ^9^ - ao desviar a atenção clínica, sobretudo na APS, para os usuários mais jovens, saudáveis e menos pobres, que têm mais condições de procurarem e se preocuparem com prevenção, dificultando o acesso e o cuidado aos mais doentes, idosos e pobres ^10^ ^,^ ^11^ ^,^ ^12^ (adoecem mais e mais gravemente, têm menos condições de se preocupar e prevenir), os quais enfrentam a precariedade e o subdimensionamento crônicos da APS e do Sistema Único de Saúde (SUS) ^13^. Vale lembrar que, em geral, o adoecimento/sofrimento presente deve ter prioridade sobre o bem-estar futuro (prevenção) ^14^.

Há um excesso de práticas preventivas mal fundamentadas que produz grande iatrogenia evitável e reforça o imperialismo preventivista ou *healthism* ^15^ ^,^ ^16^ (tendências, crenças, valores e práticas que enfatizam obrigações das pessoas buscarem a saúde e evitarem doenças ou riscos), associado à medicina de vigilância ^3^ ^,^ ^17^. Embora a prevenção seja consensualmente relevante, não parece desejável fomentar em profissionais e gestores uma atitude de repetição vaga, genérica e incondicionalmente otimista dessa mensagem. Não encontramos estudos empíricos brasileiros, mas uma revisão de literatura mostrou que profissionais têm expectativas exageradas sobre a eficácia de rastreamentos (realização de testes diagnósticos em pessoas assintomáticas) ^18^.

Com a proliferação e o uso aumentado de ações clínicas de P1 específica, voltadas para doenças ou agravos definidos, e P2 tipo rastreamento, diagnósticas e terapêuticas, houve sua incorporação na cultura profissional e da sociedade em geral, tornando necessário que profissionais e gestores tenham uma melhor formação técnica, superem a exortação preventivista genérica e orientem ações preventivas mais específicas e bem fundamentadas. Os conteúdos sobre prevenção, embora extensos na literatura científica e nos manuais clínicos por estarem presentes em uma miríade de doenças, carecem de uma estrutura sintética organizada e articuladora de conceitos que orientem profissionais de saúde (sobretudo da APS) e gestores quanto a essas ações.

O objetivo deste ensaio é apresentar uma articulação conceitual mínima orientadora da consideração pelos profissionais e gestores, de medidas de P1 específicas e P2 tipo rastreamento. Tal articulação visa favorecer a prática da prevenção quaternária (P4), definida pelos médicos de família e comunidade como “*realizada para identificar o paciente em risco de sobremedicalização, protegê-lo de nova invasão médica e sugerir-lhe intervenções eticamente aceitáveis*” ^19^ (p. 110). A P4, ao reduzir a iatrogenia e a medicalização derivadas da prevenção (comumente excessivas e evitáveis), contribui para a humanização e a melhoria das práticas preventivas ^5^, sendo, por isso, importante e necessária.

O texto está estruturado em uma argumentação sequencial e didática, dividida em tópicos que progressivamente apresentam a articulação proposta. O Quadro 1 esquematiza os principais conteúdos apresentados.

**Quadro 1:** Ideias e conceitos necessários para boas práticas preventivas.

IDEIA CENTRAL	CONCEITOS ENVOLVIDOS	APLICAÇÃO
Distinguir entre adoecimento presente e futuro (cuidado ao adoecido *vs.* prevenção em assintomáticos)	Prevenção primária (P1) Prevenção secundária (P2)	Distinga cuidado ao adoecido de P1 e P2. Confusão é comum em condutas clínicas, especialmente em doenças crônicas
Considerar o potencial de danos das intervenções preventivas	Prevenção redutiva Prevenção aditiva	Prefira prevenção redutiva criteriosa (redução do risco sem intervenções artificiais). Em caso de prevenção aditiva, o otimismo preventivo deve ser substituído por ceticismo resistente às intervenções
Considerar que tipo de estratégia preventiva está envolvida	Abordagem populacional Abordagem de alto risco	Prefira abordagem populacional, de preferência via prevenção redutiva
Usar a medicina baseada em evidências (MBE) na prevenção aditiva	*Disease oriented evidence* (DOE) *Pacient oriented evidence that matters* (POEM)	Em caso de prevenção aditiva, exija evidências de resultados finais tipo POEM e analise o balanço danos *vs.* benefícios
Escrutinar a MBE e abrir sua caixa preta, se necessário	MBE e sua crise	Escrutine as evidências para além dos *guidelines* e forças-tarefas preventivas, se houver dúvida ou polêmica razoável
Resistir ao intervencionismo na prevenção aditiva com precaução, ceticismo e cientificismo	Princípio da precaução	Em caso de persistência de dúvida razoável sobre o balanço danos *vs.* benefícios, por precaução, não oriente a medida preventiva aditiva
Estimular a autonomia e a participação e proteger da iatrogenia	Promoção da saúde, abordagem ampliada e centrada na pessoa e decisão compartilhada	Fomente o empoderamento das pessoas e coletividades para evitar danos iatrogênicos

Fonte: elaboração própria.

## Distinguir entre cuidado aos adoecidos e prevenção

Há uma tendência atual de se alterar critérios diagnósticos e com isso amplificar o que pode ser considerado patológico e diagnosticável como doença (e assim tratado), reduzindo a faixa da normalidade e reclassificando faixas de risco cada vez menores como de alto risco. Outra tendência é a incorporação de estados de alto risco nas definições de patologias e síndromes ^20^, empurrando para dentro das doenças o que era normal ou alto risco, que passa a ser manejado como patológico.

Adicionalmente, há significativos avanços tecnológicos na detecção de variações ou alterações estruturais ou funcionais mínimas ^21^. Assim, detecta-se anormalidades e disfunções cada vez menores, o que torna os prognósticos mais duvidosos e abre dúvidas sobre se a diagnose e o tratamento em fase assintomática ou precoce compensam. Um exemplo é o fenômeno do sobrediagnóstico e sobretratamento, adiante comentado. Esses processos tendem a tornar cada vez mais difícil a distinção entre prevenção (P1 e P2) e o cuidado dos que se sentem adoecidos ^12^.

Ocorre que o contrato entre profissional e usuário difere nas situações preventivas em comparação às de cuidado aos que se sentem doentes ^20^. A relação de equilíbrio sempre buscado entre os quatro valores ou princípios bioéticos clássicos (beneficência, não-maleficência, justiça e respeito à autonomia) ^22^ tende a ser significativamente diferente nessas situações devido às singularidades quanto à tolerância aos danos, ao manejo da incerteza, à fundamentação das ações e à garantia de benefícios, que exigem distintas atitudes dos profissionais e gestores ^20^ ^,^ ^23^.

No caso do cuidado clínico ao que se sente adoecido, a presença do sofrimento e dos sintomas impõe um contrato curativo ^24^ que envolve certa proatividade, em que a beneficência é fortemente valorizada e a não maleficência é comumente relativizada em função dos benefícios do tratamento (próximos no tempo), tornando aceitável um manejo flexível da incerteza e uma maior tolerância aos danos iatrogênicos. Tal manuseio é respaldado pelo estado da arte do saber e das técnicas disponíveis, não se exigindo garantia de benefício, mas ações técnica e eticamente “corretas”. Essa correção está referida ao conjunto dos saberes profissionais teóricos e técnicos vigentes, nos quais se projeta grande confiança, estendida também à experiência dos profissionais ^20^.

Tudo isso é diferente na P1 específica e nos rastreamentos (P2): não há adoecimento e sofrimento sentidos. A princípio, as pessoas estão saudáveis. O potencial de benefícios está projetado no futuro e restrito a uma minoria que adoeceria dos problemas que se busca prevenir. Nessa circunstância, a não-maleficência comumente é mais valorizada e rigorosa ^25^ ^,^ ^26^ ^,^ ^27^ ^,^ ^28^. O *primum non nocere* não pode ser relativizado pelo potencial de benefício imediato da intervenção do mesmo modo que no cuidado ao adoecido. Enquanto danos e benefícios no cuidado ao adoecido incidem na mesma pessoa, facilitando a decisão esclarecida de aceitar o tratamento, na P1 específica e nos rastreamentos o potencial de benefícios incidirá sobre uma parcela pequena das pessoas (as que adoeceriam no futuro), enquanto o potencial de danos se dissemina no presente e no futuro em todos que recebem a intervenção. Nesse caso, a compensação dos danos pelos benefícios não existe em muitas pessoas prejudicadas, e o princípio da não-maleficência é cruamente violado. Não está claro, eticamente, que o padrão de relação clínica e de consentimento dos usuários possa ser transferido da situação de cuidado ao que sente doente para a P1 e a P2, inclusive porque, ao oferecer medidas preventivas, os profissionais induzem implicitamente a aceitação das mesmas pelos usuários ^28^ com seu poder e autoridade.

Logo, a postura genericamente otimista, a atitude proativa e relativamente tolerante ao intervencionismo e à iatrogenia, e a flexibilidade para com a incerteza - típicas da clínica do adoecido - devem ser substituídas, em P1 e P2, por uma atitude cética resistente ao intervencionismo, com manejo mais rígido da incerteza. Para tal resistência atitudinal à intervenção preventiva ser superada, devem ser exigidos estudos científicos experimentais rigorosos, mostrando os resultados empíricos da aplicação de uma medida preventiva e confrontando benefícios com danos. Apenas um balanço amplamente favorável deve vencer a resistência e pender a decisão a favor da intervenção, sempre que houver um razoável potencial de danos justificador dessa cautela ^29^ ^,^ ^30^.

Devido a essas diferenças, para viabilizar boas práticas preventivas é necessário que a diferenciação entre cuidado ao que se sente adoecido e P1 (específica) e P2 (tipo rastreamento) seja valorizada e realizada no cotidiano clínico (e pelos gestores) ^20^. Ambas as situações podem ocorrer em uma mesma consulta, a serem manejadas diferentemente. Reconhecida uma situação de P1 específica ou P2 tipo rastreamento, há que abordar os danos potenciais das intervenções.

## O potencial de danos das medidas preventivas

Geoffrey Rose ^31^ chamou de medidas preventivas “*redutivas*” as ações que removem ou reduzem “*alguma exposição artificial, de modo a restaurar um estado de normalidade biológica*” ^32^ (p. 148). Trata-se de restaurar a normalidade biológica, vista como “*as condições para as quais somos considerados geneticamente adaptados devido a nossa história evolutiva*” ^32^ (p. 148), tornando as condições ambientais e os modos de vida favoráveis à saúde.

Tais medidas se concretizam em aconselhamento clínico e em ações de saúde pública e organização social: redução do sedentarismo, do tabagismo e dos alimentos multiprocessados; eliminação dos agrotóxicos nos alimentos; universalização do saneamento básico; redução das desigualdades de renda ^33^ etc. Embora as medidas preventivas redutivas possam significar mudanças no modo de viver, elas não são artificiais. Ao contrário, diminuem artificialismos patogênicos tornados banais nas sociedades moderna ^32^.

Muitas dessas medidas são relativamente aproblemáticas quanto à fundamentação científica da sua recomendação, sendo consideradas seguras (nulos ou mínimos riscos) e com presunção de benefício razoável cientificamente aceita ^32^. O relativo consenso sobre elas sustenta uma postura afirmativa e otimista, que inclusive torna prescindível a exigência de evidências científicas de alta qualidade e hierarquia (ensaios clínicos aleatorizados) sobre seus resultados. Seria inviável e antiético realizar um ensaio clínico em que o grupo controle seria exposto ao tabagismo, enquanto o grupo intervenção ficaria livre do tabaco, pois o consenso dos estudos observacionais a respeito é muito forte.

Por outro lado, Rose chamou de medidas preventivas “*aditivas*” às ações que introduzem ou “*adicionam*” no ser humano, na sua alimentação ou no meio ambiente um fator artificial, protetor e preventivo não existente na economia-fisiologia-ecologia das pessoas: vacinas, fármacos preventivos (hipotensores, hipolipemiantes), complementos alimentares artificiais (ou naturais em doses artificiais), rastreamentos etc. Essas medidas não podem ser consideradas seguras porque têm grande potencial iatrogênico, que deve ser criteriosamente avaliado. Por isso, tais ações exigem evidências de que sua realização produz resultados significativamente benéficos com nulos ou mínimos danos ^32^. Somente um balanço benefícios-danos amplamente favorável, obtido da convergência de estudos experimentais de intervenção de alta qualidade (ensaios clínicos aleatorizados) e idoneidade (pouco ou nenhum conflito de interesse) revisados, pode gerar fundamentação para sua recomendação ^34^.

Nesse balanço não devem ser aceitas justificativas teóricas ou resultados intermediários (substitutivos) aos desfechos clínicos finais (mortalidade, morbidade, qualidade de vida), porque é justificável e necessária uma desconfiança do saber teórico e da experiência dos profissionais, diversamente da situação de cuidado ao já adoecido. Como já dissemos: é necessária a avaliação dos resultados empíricos da aplicação da medida preventiva em estudos experimentais ^35^.

A distinção entre ações preventivas redutivas e aditivas é um divisor de águas que gera marcada preferência pelas primeiras, cuja segurança e benefícios são relativamente consensuais, facilitando sua recomendação por várias razões: sua segurança e eficácia; sua convergência com ações de promoção da saúde, com impacto benéfico individual e coletivo em determinantes gerais e sociais da saúde-doença; e seu caráter econômico (menor custo), sustentável e ecológico ^36^.

Por outro lado, a mesma distinção dificulta a recomendação das ações aditivas, cujo grande potencial de danos exige muita cautela. Tal cautela está escasseando na sociedade e entre os profissionais devido à socialização disseminada há décadas de várias dessas medidas, ao persistente encantamento com o desenvolvimento tecnológico (em boa parte enganoso ^35^) e à idealização ingênua da medicina baseada em evidências (a que retornaremos). Isso torna importante que essa distinção seja valorizada e exercida na prática clínica e sanitária. Ante uma medida preventiva aditiva, cabe observar que tipo de estratégia preventiva está envolvida.

## Considerar a estratégia preventiva: populacional ou de alto risco

**Figura 1:**
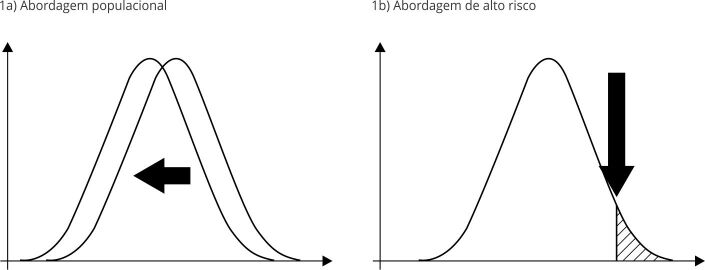
Estratégias preventivas segundo Geoffrey Rose.

Rose ^32^ discutiu dois tipos de estratégias preventivas: abordagem populacional e abordagem de alto risco. A primeira é usada em situações em que fatores de risco conhecidos são distribuídos universalmente na população. Ela consiste em intervir na população toda, visando reduzir (deslocar para a esquerda, na Figura 1a) toda a curva de risco.

Fonte: elaboração própria.

Quando toda a população usa cinto de segurança, bebe água tratada, recebe vacinas na infância, aprende a ler e escrever, e são proibidos: o fumo em lugares fechados, a bebida alcoólica ao dirigir e a propaganda de tabaco e bebidas alcoólicas, a sociedade está recebendo medidas preventivas em abordagem populacional. Se todos comem alimentos sem agrotóxicos e com menos sal, não padecem de privação socioeconômica, têm estímulo à mais atividade física em ciclovias e belas áreas verdes de lazer, via políticas de infraestrutura urbana e mobilidade sustentável, são reduzidos riscos e pode haver alto impacto na redução da morbimortalidade coletiva.

Várias dessas medidas são redutivas e outras aditivas. As redutivas incidem em determinantes gerais da saúde-doença, e sua aplicação demanda amplo apoio social/político e leis e políticas públicas difíceis de obter. Porém, uma vez efetivadas, são sustentáveis e incorporadas na vida social. Por esses motivos, Rose ^32^ defende uma ampla preferência e prioridade para essa estratégia, via ações preventivas redutivas, seguras, baratas e eficazes, por elas terem o potencial de universalizar parte importante do cuidado preventivo à saúde, concretizando com segurança esse direito humano fundamental e de cidadania.

Todavia, a estratégia que vem sendo cada vez mais aplicada é a abordagem de alto risco: identifica-se uma fração populacional de maior risco e sobre ela são aplicadas ações preventivas, sem abordar o restante da população (Figura 1b). Isso faz sentido para profissionais e usuários, que entendem o porquê da intervenção. É custo-efetiva, pois os recursos preventivos são direcionados aos com maior risco, e se encaixa no cotidiano dos serviços de saúde, que operacionalizam as ações manejando as pessoas de alto risco como doentes crônicos ^37^.

Contudo, essa estratégia tem desvantagens significativas: (1) medicaliza a prevenção; (2) precisa ser mantida indefinidamente, pois não se intervém nos determinantes gerais sociais, econômicos e culturais (sendo, por isso, cara); (3) é difícil quantificar seu real benefício à pessoa, visto que opera no campo da probabilidade; (4) gera pequeno impacto positivo na morbimortalidade, pois o pequeno grupo com alto risco produz um número bem menor de doenças e mortes do que o restante da população, de baixo risco mas muito mais numerosa; e (5) é inadequada comportamentalmente, pois exige que a pessoa de alto risco adquira novos hábitos de vida, distantes de seu entorno familiar, cultural e social, o que demanda atitudes heroicas difíceis ou inviáveis devido a iniquidades socioeconômicas e fatores psicossociais. Geralmente, tais medidas frustram médicos e usuários e culpabilizam indevidamente os últimos ^32^.

Todavia, em vez de restringir essa estratégia, tais limitações e desvantagens têm sido enfrentadas com a sua intensificação por meio do deslocamento dos pontos de corte para a esquerda (Figura 1b). Isso é o paraíso dos lucros da indústria farmacêutica e acentua ainda mais suas desvantagens, ao converter maiores proporções da população em pacientes crônicos frustrados, preocupados, vitalícios e com resultados precários ^38^. Logo, gestores e profissionais devem preferir abordagens populacionais via medidas redutivas ^39^.

Tem sido comum associar ambas as estratégias com ambos os tipos de medidas preventivas (aditivas e redutivas). A abordagem de alto risco é mais efetiva se acompanhada da abordagem populacional, mais poderosa. Um exemplo é o programa brasileiro de redução do tabagismo, exitoso (prevalência reduziu de 34% em 1989 para 14,8% em 2011) e que usou ambas estratégias e tipos de prevenção ^40^. Independentemente da estratégia, devemos avaliar o balanço benefícios-danos perante medidas preventivas aditivas, para o que outra distinção é importante e útil.

## Evidências DOE e POEM

A partir da década de 1990, intensificou-se a chamada medicina baseada em evidências (MBE). Uma das suas propostas centrais foi que se deve aproveitar na clínica dos estudos científicos sobre resultados das intervenções médicas e em saúde. Devemos migrar de uma fundamentação das decisões clínicas antes assentada na fisiopatologia, no saber acumulado pelos especialistas e seus consensos e na formação e experiência dos profissionais, para agregar a essas bases outra importante fonte de evidências: os estudos de intervenção (e observacionais) sobre os resultados clínicos das intervenções.

No topo da hierarquia das evidências ficaram os ensaios clínicos controlados aleatorizados e suas revisões sistemáticas e metanálises. Estas últimas comparam vários estudos similares nos seus resultados clínicos e produzem um conhecimento inacessível via casuísticas de profissionais e serviços ou pela análise de ensaios individualmente. O aumento da literatura científica sobre as pesquisas clínicas produziu o que foi chamado de selva da literatura médica ^41^. Um profissional clínico não tem tempo e não consegue se manter atualizado sobre o que é publicado, mesmo com a internet.

Para contribuir na seleção do que deve ser priorizado para leitura, Shaughnessy et al. ^41^ e Slawson et al. ^42^ propuseram uma distinção entre dois tipos de evidências, que chamaram DOE (do inglês *disease oriented evidence*) e POEM (do inglês *pacient oriented evidence that matters*). As evidências tipo DOE são o universo das publicações sobre as doenças: seus mecanismos fisiopatológicos, epidemiologia, técnicas diagnósticas, mecanismos de ação das terapêuticas, resultados dos tratamentos sobre os parâmetros fisiopatológicos etc. Esse conjunto é a grande maioria do saber médico, mas, embora seja a base da abordagem biomédica, não é ele o conhecimento mais importante nem para os clínicos nem para as decisões preventivas.

O que mais importa para a prevenção são os resultados das ações para as pessoas, ou seja, quanto à morbidade, qualidade de vida e mortalidade ^43^. Essas são as evidências POEM, as únicas que devem ser usadas na avaliação do balanço benefícios-danos na prevenção aditiva. Além de estritamente necessárias, elas são uma pequena proporção da literatura. É crucial que os desfechos avaliados nesses estudos sejam finais, ou seja, que interessem às pessoas (POEM) e incluam os danos. Isso é importante porque frequentemente são usados desfechos intermediários (ou substitutos) nos ensaios clínicos, que exigem suposições teóricas para sua valorização. Há esforços em mensurar a ligação empírica entre desfechos finais e intermediários para fundamentar a aceitação de evidências sobre desfechos intermediários como prova de eficácia ^44^. Todavia, a peculiaridade da prevenção em suas diferenças já citadas não é reconhecida e considerada nessas discussões. A grande valorização da não-maleficência em assintomáticos sustenta que a prevenção deve ser destacada e desfechos intermediários não devem ser aceitos nela.

Se uma ação preventiva aditiva é usada há tempos, a permanência de sua recomendação exige avaliação periódica baseada em revisões sistemáticas dos ensaios clínicos e também dos estudos observacionais nas populações. Esses últimos estão abaixo na hierarquia das evidências, mas são muito importantes nas medidas preventivas já em uso e na avaliação da eficácia e segurança (poucos danos). Isso implica uma maior aproximação para com a MBE.

## A MBE e a sua crise

A MBE progressivamente se impôs como um novo regime de poder epistemológico e cultural sobre as práticas médicas e sanitárias ^45^. Deslocou em grande parte o poder de decisão clínica dos profissionais (suas escolas e experiências acumuladas) para um novo circuito de legitimidade e discussão que envolve a literatura científica (ensaios clínicos, revisões sistemáticas e metanálises), bem como as instituições produtoras de revisões e diretrizes clínicas baseadas em evidências.

Como consequência, a MBE ganhou um novo poder que atravessa sociedades e culturas locais, ignora as experiências dos profissionais e tende a se impor como uma superior norma de excelência técnica, com inédita legitimidade científica. Ela induz uma padronização das condutas clínicas, produzindo, paradoxalmente, um potencial efeito contrário ao aperfeiçoamento das práticas médicas: o fortalecimento de uma medicina burocrática ^46^, via aplicação de protocolos/diretrizes baseados nas revisões/metanálises.

Indústrias farmacêuticas, capazes de financiar ensaios clínicos e metanálises e influenciar os especialistas que se envolvem nisso, tornaram-se atores dominantes e com poder incomparável sobre a produção do saber médico, agora produção industrial ^47^. Apesar das boas intenções e de vários sucessos relevantes, a MBE tem limitações e já foi identificado uma crise em seu interior, devido aos valores ^48^ e interesses que a atravessam. Trata-se do afastamento de sua proposta original na direção de uma nova governança aberta a interesses escusos. A MBE foi apropriada por interesses das indústrias farmacêuticas e de equipamentos médicos, o que torna a sua confiabilidade uma ilusão ^49^.

O próprio aumento de exigências de maior qualidade nos ensaios clínicos e revisões, a fim de sanar os vieses dos estudos patrocinados pelas indústrias, aumenta o seu custo e os torna paradoxalmente mais dependentes delas ^50^. O volume de diretrizes clínicas se tornou enorme, sendo impossível para o clínico, de novo, se atualizar. Benefícios estatisticamente significativos podem ser marginais na prática clínica, mas vem com a etiqueta da MBE. Regras inflexíveis e diretrizes via MBE podem produzir cuidados orientados pelo gerenciamento em vez de centrados na pessoa; elas mapeiam mal a multimorbidade complexa comum nos usuários da APS ^51^ ^,^ ^52^ ^,^ ^53^.

As incertezas e problemas são tantos que foi proposto o conceito de “incerteza médica ampla” (BMU, do inglês *broad medical uncertainty*) para descrever a situação da desconfiança no saber médico atual ^54^. Particularmente importante é a situação já apontada de opacidade dos dados, fechados ao escrutínio independente, apesar do quase consenso e das conclamações sobre a necessidade de compartilhamento dos dados primários para melhorar a confiabilidade ^55^. Apesar disso, a comunidade médico-científica vem tolerando que sejam mantidos ocultos os dados primários de pesquisas financiadas pelas indústrias, inacessíveis aos pesquisadores independentes.

Essa digressão sobre os problemas da MBE visa fundamentar que a avaliação das evidências sobre tal ou qual medida preventiva aditiva necessita (não raramente) ir além da consulta aos portais de evidências disponíveis, da Colaboração Cochrane (mais respeitada instituição produtora de revisões sistemáticas), das forças tarefas nacionais de serviços preventivos e das diretrizes institucionais nacionais. Isso não significa que não se deva aproveitar essas fontes, mas elas não devem ser o final da avaliação. Em vários e relevantes casos, devem ser o começo.

Havendo evidências confiáveis (poucos ou nenhum conflito de interesse) e de boa qualidade, se o balanço benefícios-danos apresentar amplo benefício líquido com poucos danos, uma rápida consulta digital confirmará o consenso da recomendação ao mostrar um nível de evidência alto e/ou força de recomendação forte (usando o GRADE - *Grading of Recommendations, Assessment, Development, and Evaluations* ^56^ ^,^ ^57^) ou um grau de recomendação A, na classificação da Força-tarefa dos Serviços Preventivos dos Estados Unidos (USPSTF) ^58^, para a medida preventiva em questão. Porém, em casos relevantes, há grau de recomendação B, que indica precária qualidade das evidências e ou polêmica nas suas interpretações, merecendo, então, um estudo mais cuidadoso. Dois exemplos de alta relevância sanitária ilustram essa situação.

O rastreamento mamográfico de câncer de mama é recomendado por todas as diretrizes preventivas nacionais ocidentais (salvo da Suíça), mas com grau B de recomendação (pela USPSTF). Há polêmica intensa na literatura especializada, com menos benefícios do que anteriormente estimado, vários estudos observacionais de boa qualidade mostrando pouco e mesmo nenhum benefício, e danos graves (os sobrediagnósticos/sobretratamentos, principalmente) ^59^ ^,^ ^60^.

As diretrizes baseadas em evidências sobre o uso em P1 das estatinas (drogas redutoras do colesterol sanguíneo), incorporadas nos manuais e na prática clínica, tinham sérios problemas de conflitos de interesse e mostram pequeno benefício e total opacidade dos dados primários sobre os efeitos adversos, inviabilizando um confiável balanço benefícios-danos ^61^ ^,^ ^62^. Na metanálise que embasou a recomendação de uso de estatinas em P1, de 2012 ^63^, todos os autores dos ensaios metanalisados foram em grande parte ou totalmente financiados por indústrias farmacêuticas ^62^. O grupo que realizou a metanálise (financiado pelas mesmas indústrias) não teve acesso à totalidade dos dados primários sobre os efeitos adversos, e nenhum outro grupo teve acesso a quaisquer dados primários, mantidos em sigilo industrial ^61^. A situação duvidosa/opaca ou de dissenso científico nesses dois casos, envolvendo potencialmente ou concretamente danos graves/extensos, justifica precaução (a seguir detalhada).

Localizada uma medida preventiva aditiva duvidosa, uma busca adicional em periódicos científicos de alta qualidade pode revelar as polêmicas e seus fundamentos. Nos dois casos acima, artigos no *BMJ* esclarecem e dirigem uma análise crítica da questão, às vezes remetendo a outros artigos e periódicos. Pesquisadores independentes sintetizam os problemas, e uma consideração cuidadosa pode/deve ser realizada. Pode ser o caso de se agir contrário às diretrizes clínicas e/ou institucionais para se manter a excelência do cuidado preventivo e proteger os usuários. Nesses casos críticos, os conceitos antes apresentados enriquecem a avaliação. Porém, um conceito (e prática) já bem desenvolvido, mas pouco utilizado na medicina preventiva, é especialmente adequado e útil nos casos duvidosos: o princípio da precaução (PP).

## Resistir à prevenção aditiva duvidosa: o princípio da precaução

O PP nasceu na Europa nos anos 1970, no contexto da crise ecológica. Ele se expandiu e se consolidou no direito ambiental devido à necessidade de se tomar providências diante de perigos ecológicos de grande monta, na vigência de dúvidas científicas ou de polêmicas sobre as causas desses perigos (chuva ácida, diminuição acentuada dos peixes, aquecimento global, buraco na camada de ozônio etc.). O PP orienta que, ante o perigo de danos extensos e graves ao ambiente e às pessoas e mesmo havendo dúvidas científicas sobre as causas, os governos e agências reguladoras devem agir para proteger do dano: “*...a ausência de absoluta certeza científica não deve ser utilizada como razão para postergar medidas eficazes e economicamente viáveis para prevenir a degradação ambiental*” ^64^ (p. 157).

Tesser & Norman ^36^ sintetizaram a operacionalização do PP em cinco componentes: (1) evitar ativamente o dano gerado por atividade ou produto em face da incerteza; (2) inverter o ônus da prova sobre a atividade suspeita: são os seus defensores que devem provar sua eficácia e segurança; (3) explorar alternativas inofensivas para os mesmos fins da atividade suspeita; (4) aumentar a participação pública na tomada de decisão; e (5) monitorar ativamente o estado do conhecimento científico sobre o problema, pois novas evidências podem mudar sua avaliação.

Há discussões a favor e contra e versões distintas do PP. Uma proposta que o entende como uma regra de decisão destacou o sentido comum de várias versões do princípio, por meio do chamado “tripé de decisão”: (1) uma condição de dano (D), que especifica uma ameaça de dano catastrófico que deve ser evitado; (2) uma condição epistêmica (E), indicando que a probabilidade desse dano ocorrer não é desprezível e existem bons fundamentos epistêmicos para levar a ameaça a sério; e (3) um remédio sugerido (R), que recomenda medidas para evitar a catástrofe ^65^ ^,^ ^66^ ^,^ ^67^. Em outras palavras: se um resultado previsto é considerado prejudicial (D) e a perspectiva de que o dano se materializará é suficientemente plausível (E), então medidas cautelares (o remédio sugerido) devem ser tomadas (R) ^68^.

Steel ^69^ ^,^ ^70^ acrescentou dois componentes ao tripé, restringindo sua aplicação: (1) a regra da proporcionalidade, em que as medidas de precaução devem ser calibradas para o grau de incerteza e a gravidade das consequências temidas: o “remédio” não deve ser pior do que a “doença”, e os efeitos colaterais negativos das medidas de precaução devem ser reduzidos ao mínimo; e (2) o princípio da metaprecaução, em que a incerteza científica não deve levar à paralisia na tomada de decisões diante de uma ameaça de dano grave.

Hopster ^68^ propôs a regra da ligação inversa: quanto maior a catástrofe prevista, menos evidências são necessárias para desencadear uma ação cautelar para evitá-la; quanto menor a catástrofe, mais evidências são necessárias para justificar a precaução. Essa regra só entra em jogo se as evidências de risco de danos existirem e forem minimamente plausíveis, razoáveis, o que serve para evitar a “paranóia da precaução”: devemos ser capazes de ignorar riscos suficientemente improváveis.

Em outra abordagem, Sandin & Peterson ^71^ defendem que o PP é um princípio moral de nível médio que pode ser considerado como os outros quatro princípios de Beauchamp & Childress ^22^, comuns nas discussões clínicas e sanitárias: beneficência; não-maleficência; justiça; e respeito à autonomia. O PP seria um quinto princípio a ser ativado nas circunstâncias discutidas, sendo “adicionado” aos anteriores.

Por seu grande potencial de danos, as medidas preventivas aditivas (P1 específica e P2 tipo rastreamento) são candidatas naturais a serem escrutinadas pelo PP ^36^ em situações de incerteza sobre o balanço danos-benefícios (já demandado usualmente na medicina e saúde pública) devido à forte valorização da não-maleficência.

Esse balanço pode ser duvidoso, sendo possível haver polêmica na interpretação das evidências e/ou dúvidas científicas dificultando uma conclusão consensual. Porém não é necessário um consenso sobre a dimensão dos benefícios e danos ou dobre o seu balanço para uma decisão bem fundamentada. A existência de polêmica científica razoável sobre tal - ou a ausência de consenso sobre uma ampla margem de benefícios líquidos com poucos danos, tornando o balanço duvidoso - demanda aplicação do PP para se evitar danos iatrogênicos graves e/ou extensos. Uma primeira operacionalização de medida corretiva é teoricamente simples e fácil: não implantar a medida preventiva ou inverter a recomendação para sua realização, se já em uso.

Na P1 específica e nos rastreamentos, geralmente todos os envolvidos compartilham dos mesmos interesses preventivos, não havendo conflitos explícitos - diferentemente da quase totalidade dos contextos de aplicação do PP. Profissionais, gestores dos sistemas de saúde, governos, agências reguladoras e usuários almejam a prevenção de doenças, mortes e sofrimentos evitáveis. Se uma ação preventiva aditiva já é usada rotineiramente, ela foi aprovada e recomendada por instituições governamentais, científicas e ou profissionais (saúde pública, agências reguladoras, saber científico, associações de especialistas). Logo, se cabível, a primeira providência por precaução para evitar o seu dano iatrogênico é a não aprovação da ação preventiva, ou a suspensão da sua recomendação positiva se já em uso.

Propomos a seguinte formulação para o PP na prevenção: a ausência de certeza científica de que uma medida preventiva aditiva proporciona amplos benefícios líquidos com poucos danos iatrogênicos deve ser suficiente para a não implantação ou suspensão de tal medida. Isso permite evitar danos iatrogênicos sem problemas de proporcionalidade, pois a suspensão ou inversão da recomendação não tem custos ou efeitos colaterais negativos.

Devido à inversão do ônus da prova, a discussão de um uso mais restrito ou rigidamente regulado de uma medida aditiva de P1 específica ou P2 tipo rastreamento, que foi recusada ou suspensa em aplicação mais generalizada, deve constituir um novo processo de avaliação da medida, então proposta para outro uso/contexto.

## Promover participação, empoderamento e decisão compartilhada

Finalizada a avaliação sobre tal ou qual medida preventiva, restará promover no contexto clínico com pessoas concretas a participação do usuário, seu empoderamento e uma decisão compartilhada. Não há espaço aqui para tratar desses temas complexos, cruciais para uma boa efetividade clínica e um bom relacionamento profissional-usuário, mas eles nos resgatam da elaboração cognitiva unilateral e nos devolvem ao contexto da interação clínica, que demanda: uma abordagem centrada nas pessoas ^72^; habilidades de comunicação adequadas ^73^ ^,^ ^74^ para um diálogo aberto e horizontalizado ^75^; e uma abordagem ampliada ^76^, que parta da situação psicossocial e existencial dos usuário e acesse a sua perspectiva, para com ele construir uma base comum de entendimento sobre sua situação e a ação preventiva nela contextualizada, fomentando sua autonomia ^77^ e viabilizando uma maior humanização do cuidado. A melhor proposta preventiva corre grande risco de se frustrar caso não seja contextualizada, compartilhada e pactuada com o usuário. Estando fora do alcance deste trabalho, apenas registramos e enfatizamos, sem desenvolver, a necessidade desse retorno ao relacionamento profissional-usuário e seus desafios para viabilizar boas práticas clínicas preventivas.

## Dificuldades

Vários fatores dificultam a proposta aqui apresentada. Como vimos, a presença de conflitos de interesses na produção e na interpretação das evidências é um deles. O poder dos interesses econômicos envolvidos na MBE tem enviesado e fraudado as evidências e suas interpretações, introduzindo, mudando ou mantendo intervenções preventivas ^49^ ^,^ ^78^.

Outro fator é a ampla tolerância social e cultural a danos derivados de ações preventivas, que são diluídos nos efeitos adversos comuns e largamente tolerados nas intervenções médicas ^35^. Avalizada pela biomedicina e pelo Estado, essa tolerância se disseminou e se legitimou social e culturalmente, e sempre pode ser defendida com base em sucessos prévios (a exemplo de vacinas, antibióticos etc.). Adicione-se a crença, o “estado de opinião” e o valor moral, favoráveis às ações preventivas, e se configura uma situação de grande dificuldade sociocultural e política para a postura anti-intervencionista, o ceticismo e o rigor da aplicação do PP aqui defendidos na prevenção aditiva. Tal situação facilita a introdução de novas ações preventivas aditivas, mesmo que duvidosas, por muitas vezes virem embaladas com o rótulo da MBE.

Acirrando esses fatores, há o fenômeno do paradoxo da popularidade ^21^ ^,^ ^79^: o grande apoio a algumas ações preventivas aditivas é alimentado pelos efeitos danosos de algumas delas, que não podem ser percebidos individualmente. É o caso dos sobrediagnósticos, comuns nos rastreamentos: diagnósticos corretos de doenças que não se desenvolveriam clinicamente na vida da pessoa, mas que não podem atualmente ser distinguidos de doenças que avançariam (p.ex.: o rastreamento de vários cânceres), pelo que são todas tratadas (sobretratamento).

Como o sobrediagnóstico é um fenômeno perceptível apenas nos dados epidemiológicos (coletivos e retrospectivos), os profissionais e usuários vivem uma cegueira epistêmica ^80^: todos os sobrediagnosticados se sentem salvos pela detecção e pelos tratamento precoces e propagandeiam o rastreamento, tendo sido gravemente e vitaliciamente prejudicados. Os rastreamentos são os maiores produtores de sobrediagnóstico, mas profissionais e usuários só recebem *feedback* positivo ao rastrear (das instituições e do entorno profissional e sociocultural) ^81^, mesmo quando não há um balanço benefícios-danos claramente favorável. Esse fenômeno dificulta o distanciamento crítico e o ceticismo científico anti-intervencionista aqui defendidos, afastando o PP. O sobrediagnóstico ocorre em grandes proporções nos rastreamentos de câncer de mama, próstata, pele, tireoide e rim ^82^.

Outro fator dificultador é a comum crença no avanço tecnológico, tido como sempre benéfico e poderoso. Crê-se, ingenuamente, que eventuais danos, se foram tolerados pelas agências reguladoras e pelos médicos, foram compensados por benefícios significativos, e que o desenvolvimento tecnológico vai atenuá-los ou evitá-los no futuro - veja-se as “promessas” para a prevenção do câncer ^83^, por exemplo.

Por outro lado, fatores estruturais, sistêmicos e institucionais são relevantes. Mesmo quando profissionais e usuários desejam resistir a intervenções duvidosas (não fazer, fazer menos), o sistema dentro do qual o atendimento é prestado pode tornar isso difícil ^84^. Auditorias e diretrizes clínicas geralmente induzem a fazer mais intervenções, especialmente as preventivas, e os profissionais têm dificuldade de se afastar delas ^85^. A maior consciência do potencial de reclamações e do risco de litígio torna a prática profissional mais defensiva ^86^, podendo gerar solicitação de mais exames e tratamentos “para segurança” do profissional ^87^.

Finalmente, outro fator é o poderoso apelo emocional preventivista vigente no ambiente do cuidado especializado às doenças crônicas (cardiovasculares e cânceres, principalmente), que extravasa para a APS, para os sistemas de saúde, e para a sociedade e a cultura geral. Os especialistas são referência social e técnica, com alta legitimidade sociocultural e epistêmica sobre determinadas doenças. Isso transforma sua opinião pessoal (e os consensos dos especialistas e diretrizes clínicas respectivas) em fonte potente de poder cultural, social e político, geralmente pró-intervenções preventivas.

Entretanto, diferentemente do cuidado ao adoecido, em que pequena porção das decisões é suprida pela MBE, as decisões em P1 específica e P2 aditivas “*devem ser baseadas estritamente nas melhores e mais atualizadas evidências, porque elas são a única fonte confiável de informação*” ^59^ (p. 5). Por isso, o apelo emocional, as atitudes, os saberes e as experiências desses especialistas devem ser postos em suspensão e evitados nas decisões sobre prevenção aditiva ^59^.

Todos os fatores acima citados criam expectativas ficcionais atenuadoras da incerteza do futuro, que enfatizam benefícios potenciais em vez de danos ^88^, produzindo um otimismo pró-intervencionista e moralista que dificulta a atitude crítica/cética/anti-intervencionista e a aplicação do PP. Todavia, essas dificuldades não diminuem a necessidade de redução de danos e medicalização desnecessária, melhorando as práticas preventivas.

## Considerações finais

Apresentamos um conjunto de conceitos e critérios articulados para serem usados por clínicos e gestores no manejo da prevenção primária específica e secundária tipo rastreamento. Eles inovam ao defender a introdução de um radical estranhamento e desconfiança, indutores de inéditos rigor científico e precaução (hoje inexistentes) quanto às medidas preventivas com significativo potencial de danos (ações preventivas aditivas).

As principais novidades são a valorização do divisor de águas que é a distinção entre ações preventivas redutivas (redutoras dos riscos sem intervenção artificial) e aditivas (adição de fatores artificiais de proteção) e a defesa da aplicação do princípio da precaução aos casos de prevenção aditiva duvidosos, pouco discutidas e praticadas na medicina e na saúde pública. A ausência dessa aplicação tem facilitado a introdução e a permanência de práticas preventivas duvidosas ou pouco eficazes e iatrogênicas, geradoras de iniquidades e fomentadas pela cultura biomedicalizada, pelo imperialismo preventivista e pela medicina da vigilância, em sinergia com os interesses escusos e corporativos, os argumentos da autoridade (profissional ou científica), e as manipulações da MBE ou a sua desconsideração ^89^.
